# Quantifying the Tendency of Therapeutic Target Proteins to Bind Promiscuous or Selective Compounds

**DOI:** 10.1371/journal.pone.0126838

**Published:** 2015-05-22

**Authors:** Ye Hu, Jürgen Bajorath

**Affiliations:** Department of Life Science Informatics, B-IT, LIMES Program Unit Chemical Biology and Medicinal Chemistry, Rheinische Friedrich-Wilhelms-Universität, Bonn, Germany; University of Bologna & Italian Institute of Technology, ITALY

## Abstract

The ability of target proteins to bind structurally diverse compounds and compounds with different degrees of promiscuity (multi-target activity) was systematically assessed on the basis of currently available activity data and target annotations. Intuitive first- and second-order target promiscuity indices were introduced to quantify these binding characteristics and relate them to each other. For compounds and targets, opposite promiscuity trends were observed. Furthermore, the analysis detected many targets that interacted with compounds representing a similar degree of structural diversity but displayed strong tendencies to recognize either promiscuous or selective compounds. Moreover, target families were identified that preferentially interacted with promiscuous compounds. Taken together, these findings further extend our understanding of the molecular basis of polypharmacology.

## Introduction

Polypharmacology is an emerging theme in pharmaceutical research and chemical biology based upon the premise that compounds frequently act on multiple targets [1–5], thereby triggering complex functional responses and pharmacological effects. Compound promiscuity, defined as the ability of small molecules to specifically interact with multiple targets, provides the molecular basis of polypharmacology [6,7]. On the other hand, since there are many more active compounds than targets available, polypharmacology also requires the ability of targets to specifically bind multiple (and structurally distinct) ligands. In other words, many pharmaceutically relevant proteins must be “good” small molecule targets. Otherwise, polypharmacology on a larger scale would be difficult to rationalize. An analysis of compounds active against the current spectrum of pharmaceutical targets has revealed that many targets recognize large numbers of structurally diverse compounds [8], which is well in accord with assumed ligand-target interaction characteristics underlying polypharmacology, as discussed above.

While compound/drug promiscuity has been the topic of a number of investigations and reviews [5–7], promiscuity at the target level has thus far only been little explored in a systematic manner. Compound promiscuity can be quantified by collecting available high-confidence activity/target annotations, thereby providing a conservative estimate of the degree of promiscuity [5,6]. Analogously, one might estimate target promiscuity by counting the number of known structurally distinct active compounds for a given target for which well-defined activity measurements are available. Such simple measures are sufficient to assign different promiscuity levels to active compounds and targets on the basis of currently available data or aid in the generation of compound-based target or drug-target networks. However, they do not provide any information about the potential interplay of promiscuity at the ligand and target levels.

Having studied compound promiscuity from different viewpoints [6,7], we have been interested in exploring target promiscuity taking compound promiscuity information into account. Specifically, we have asked the questions if there might be detectable tendencies for targets to either recognize promiscuous or selective compounds and how such tendencies might relate to the ability of targets to interact with increasing amounts of structurally diverse compounds. The analysis presented herein was designed to address these and related questions and has yielded in part surprising results, as detailed in the following.

## Material and Methods

### Data collection

From the latest version of ChEMBL (release 20) [9], compounds were extracted for which direct interactions (i.e., assay relationship type “D”) with human targets at the highest level of confidence (i.e., assay confidence score 9) were reported. Only “single protein” targets were considered. Two different types of potency measurements, including (assay-independent) equilibrium constants (K_i_) and (assay-dependent) IC_50_ values, were separately collected (because these types of measurements should not be directly compared). To ensure high data confidence, only explicitly defined potency values were retained. All approximate measurements such as “>”, “<”, or “∼” were discarded. Compounds with multiple K_i_ or IC_50_ values for the same target were selected if all values fell within the same order of magnitude. Then, the geometric mean of all values was calculated as the final potency annotation. In addition, only compounds with at least 1 μM potency (i.e., pK_i_ or pIC_50_ ≥ 6) were considered. Furthermore, all targets with active compounds were organized into target families following the protein classification hierarchy of ChEMBL and UniProt family annotations [10].

On the basis of these selection criteria, two activity measurement-dependent data sets were generated, including a K_i_ and an IC_50_ value-based set. If a compound was annotated with both K_i_ and IC_50_ values, it was assigned to both sets. In addition, from all qualifying compounds, molecular scaffolds were extracted by removing all side chains and retaining ring systems and linkers between them [11]. Scaffolds were isolated to represent structurally distinct compound series. In addition, scaffolds were further reduced to cyclic skeletons (CSKs) by converting all heteroatoms to carbon and all bond orders to one [12]. Hence, each CSK represented a set of topologically equivalent scaffolds.

### Assessment of target promiscuity

**Fig 1:**
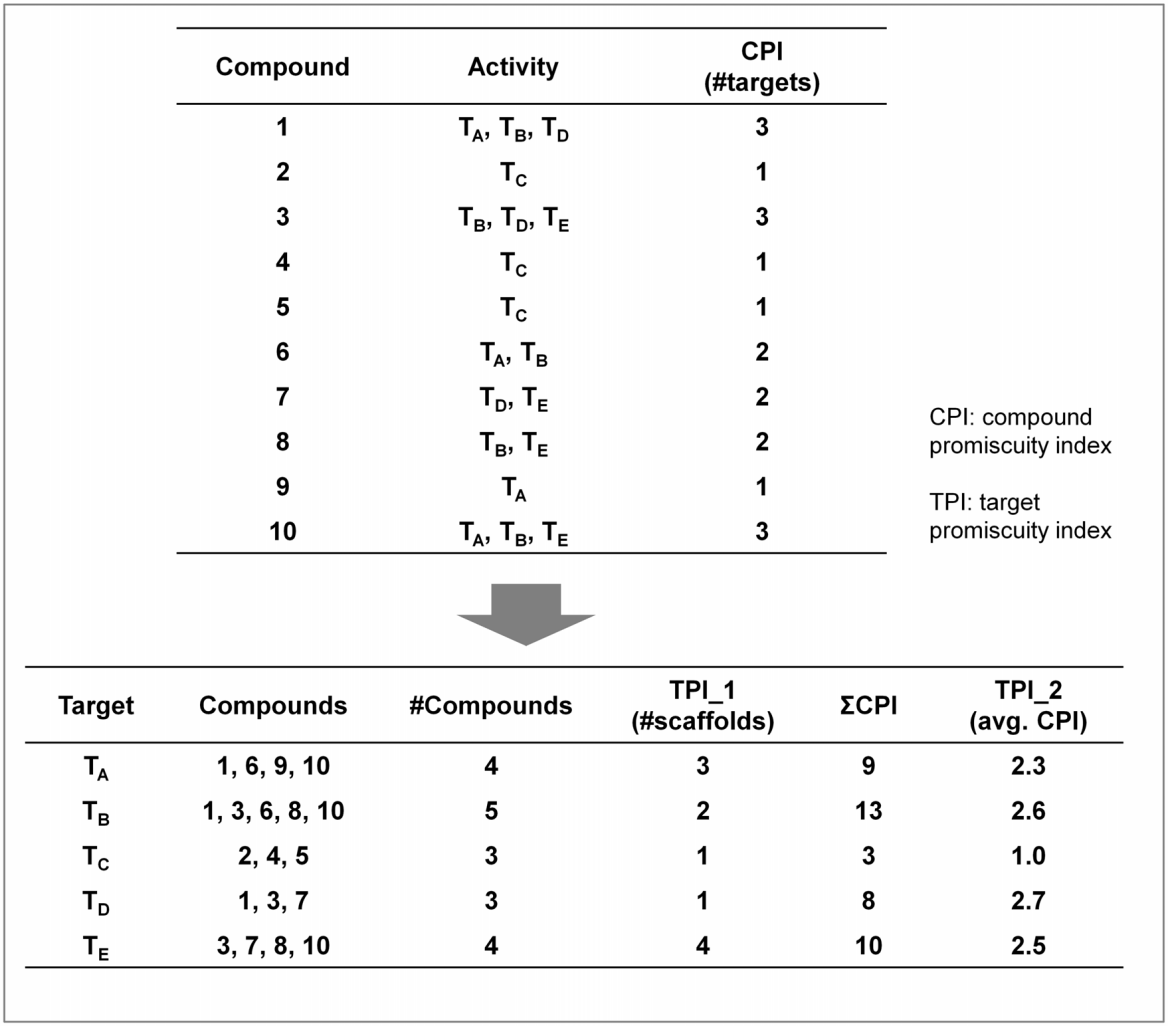
Calculation of first- and second-order target promiscuity indices. Shown is a workflow that illustrates how first- and second-order target promiscuity indices are calculated. On the basis of compound activity data, the activity profile of a compound is generated by collecting all available target annotations (top). Accordingly, for each compound, the number of targets it is active against is counted to yield the *compound promiscuity index* (CPI). Then, all compounds active against the same target are grouped (bottom). For each target, the number of unique scaffolds contained in its ligands is determined as the *first-order target promiscuity index* (TPI_1). Furthermore, CPI values of all compounds interacting with a given target are summed and the average CPI value is calculated as the *second-order target promiscuity index* (TPI_2).

To assess the degree of target promiscuity, different indices were defined, as illustrated in Fig 1. On the basis of high-confidence compound activity data assembled from ChEMBL, the activity profile of a compound was generated by collecting all available target annotations. Accordingly, for each compound, the number of its known targets was counted to yield the *compound promiscuity index* (CPI). In the example in Fig 1, compound 1 is active against three targets, yielding a CPI value of 3. Furthermore, compounds active against the same target were grouped. For example, in Fig 1, target T_A_ interacts with four compounds (1, 6, 9, 10) and target T_C_ with a distinct set of three compounds (2, 4, 5). For each target, the number of unique scaffolds representing active compounds was determined as the *first-order target promiscuity index* (TPI_1). Furthermore, CPI values of all compounds known to interact with a given target were summed and the average CPI value was calculated to yield the *second-order target promiscuity index* (TPI_2). For example, in Fig 1, the four compounds active against target T_A_ contain three unique scaffolds, resulting in a TPI_1 value of 3. In addition, these four compounds have a total of nine target annotations, yielding a TPI_2 value of 2.3 for T_A_. By contrast, compounds 2, 4, and 5 are exclusively active against T_C_, resulting in a TPI_2 value of 1 for T_C_.

## Results and Discussion

### Activity data and compound sets

Initially, we briefly summarize the results of data selection and curation and the assembly of the data sets upon which our subsequent promiscuity analysis was based.

#### Organization of compound data sets

On the basis of the data selection and curation criteria detailed above, two sets of compounds were assembled for which high-confidence activity data for human targets were available by separately considering K_i_ and IC_50_ measurements, as reported in Table 1. In this context, it is also noted that records of inactivity in target-based assays were not available for compounds selected for promiscuity analysis. The K_i_ value-based set consisted of 43,086 compounds active against 613 targets. These compounds formed a total of 67,049 compound-target interactions and were represented by 16,071 unique scaffolds and 7880 CSKs. The IC_50_ set was much larger than the K_i_ set, containing 75,244 compounds annotated with 1069 targets forming nearly 95,000 compound-target interactions. The IC_50_ set compounds yielded 28,875 scaffolds and 12,856 CSKs (Table 1).

**Table 1:** Data sets.

Number of	K_i_	IC_50_
**Compounds**	43,086	75,244
**Targets**	613	1069
**Interactions**	67,049	94,508
**Scaffolds**	16,071	28,875
**CSKs**	7880	12,856

For the K_i_ and IC_50_ value-based data sets, the number of compounds, targets, and compound-target interactions is reported. In addition, the number of unique scaffolds and cyclic skeletons (CSKs) obtained from active compounds is provided.

#### Compound, scaffold, and CSK distributions

**Fig 2:**
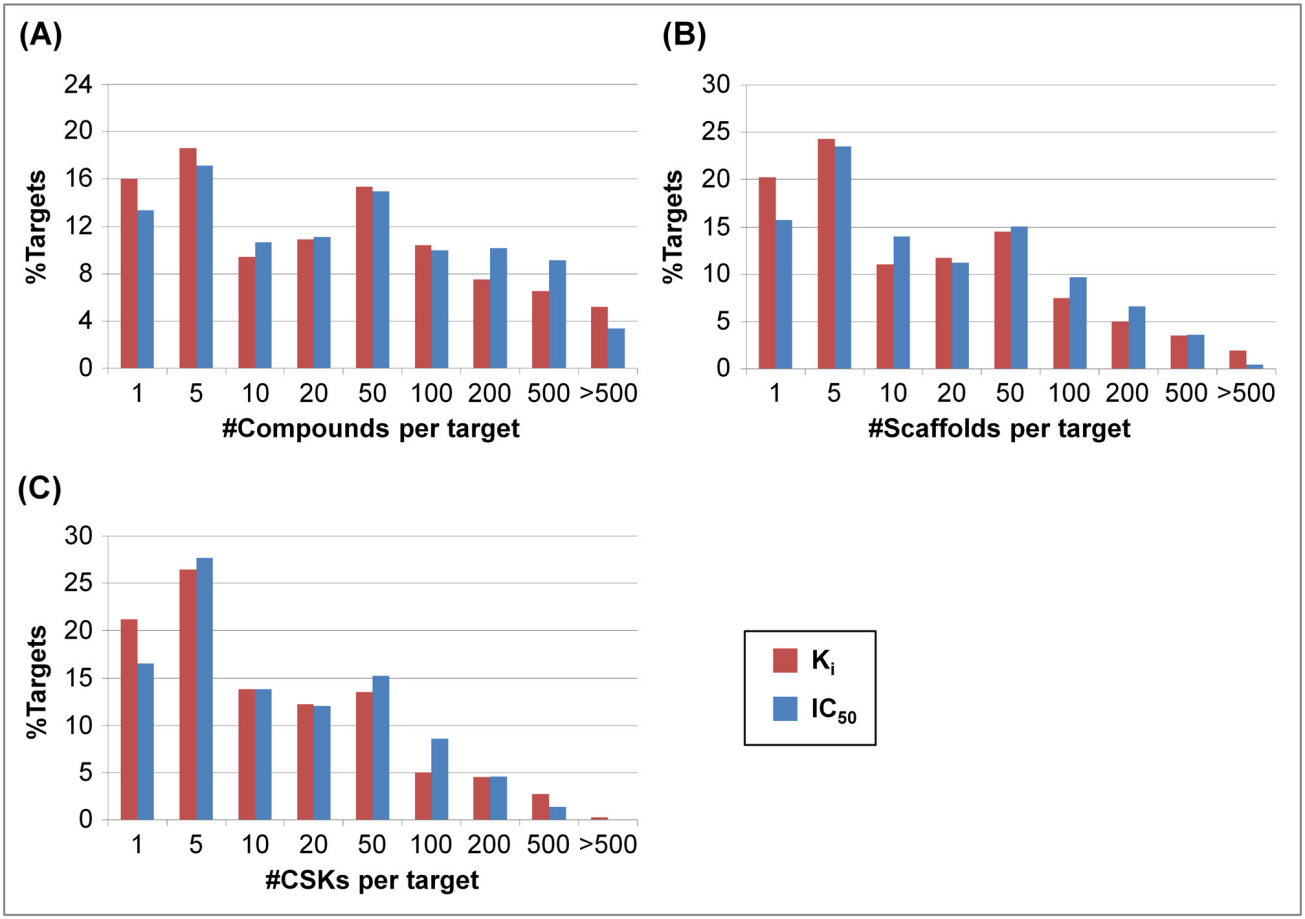
Distributions of compounds, scaffolds, and cyclic skeletons. The percentage of targets with increasing numbers of (**A**) compounds, (**B**) scaffolds, and (**C**) CSKs is reported for the K_i_ (red) and IC_50_ (blue) data sets.

Fig 2 reports the distribution of compounds, scaffolds, and CSKs over different target proteins. For ~35% (K_i_ set) and ~31% (IC_50_ set) of all targets, only one to five compounds were available, as reported in Fig 2A. For the majority of the targets, 10 or more active compounds were available. Moreover, 32 targets (i.e., ~5%; K_i_) and 36 targets (~3%; IC_50_) with more than 500 active compounds were identified. Fig 2B and 2C reveal comparable distributions for scaffolds and CSKs for the K_i_ and IC_50_ sets. For large numbers of target proteins, active compounds were found to contain one to five scaffolds or CSKs. In particular, for ~20% (K_i_) and ~16% (IC_50_) of the targets, only one scaffold or CSK was available. On average, compounds active against each target yielded 45 and 38 scaffolds and 29 and 25 CSKs for the K_i_ and IC_50_ value-based sets, respectively, reflecting the average degree of scaffold diversity across current pharmaceutical targets. Compared to the IC_50_ set, targets in the K_i_ set were generally associated with more compounds, scaffolds, and CSKs. Targets for which fewer than 10 active compounds were available were not further considered (given their low degree of exploration). The final K_i_ and IC_50_ data sets assembled for promiscuity analysis comprised 354 and 649 targets, respectively.

### Promiscuity indices

#### Concept

Different promiscuity indices were defined for our analysis, as illustrated in Fig 1. Counting the number of target annotations for a given compound yielded the *compound promiscuity index* (CPI), a standard measure for assessing the degree of compound promiscuity that is often applied [6]. Furthermore, to assess target promiscuity, two indices were defined. For each target, the number of unique molecular scaffolds from all active compounds was determined, yielding the *first-order target promiscuity index* (TPI_1). This index accounted for the ability of a target to interact with structurally diverse compounds. We note that this index did-by design- not consider the number of compounds represented by each scaffold, which would often bias the statistics. For example, if a scaffold represented 10 related active analogs, it was considered equivalent to a scaffold representing two actives. Hence, the total number of different core structures recognized by a given target was accounted for by TPI_1 (not the absolute number of compounds represented by them). In addition, CPI values of all compounds active against a target were summed and the average CPI value was calculated to yield the *second-order target promiscuity index* (TPI_2). Thus, TPI_2 accounted for the degree of promiscuity among all compounds active against the target. Accordingly, different from TPI_1, the total number of active compounds was taken into consideration in the calculation of TPI_2. The minimal value of TPI_2 was 1, indicating that all compounds active against a given target were exclusively active against this target. By contrast, a TPI_2 value of 5 would indicate that compounds active against the target were on average active against five targets. Therefore, comparison of TPI_1 and TPI_2 revealed if a target that interacted with a certain amount of structurally distinct compounds might preferentially bind promiscuous compounds (with multi-target activities) or more selective compounds. These comparison can be extended to multiple targets, for example, targets with the same or similar TPI_1 values (i.e., targets binding compounds with a comparable level of scaffold diversity) or entire target families. For example, in Fig 1, targets C and D interact with compounds represented by a single scaffold, thus yielding the same TPI_1, but different TPI_2 values (i.e., 1.0 vs. 2.7) because these active compounds have different promiscuity.

We also note that the conventional CPI definition applied here does not take into account if targets of promiscuous compounds are related to each other or not. However, it has recently been shown that only ~2% of bioactive compounds are promiscuous across different unique target families on the basis of high-confidence activity data (as used herein) [7]. Thus, most promiscuous compounds act on related targets, as quantified by CPI calculations. This also has implications for the consideration of other possible compound promiscuity measures. For example, one could envision introducing a CPI variant to account for activity against unique target families, rather than individual targets. However, given the very low promiscuity rate across different families, most values of this CPI variant would be one (and hence not suitable for TPI_2 calculations).

#### Distribution of promiscuity indices

**Fig 3:**
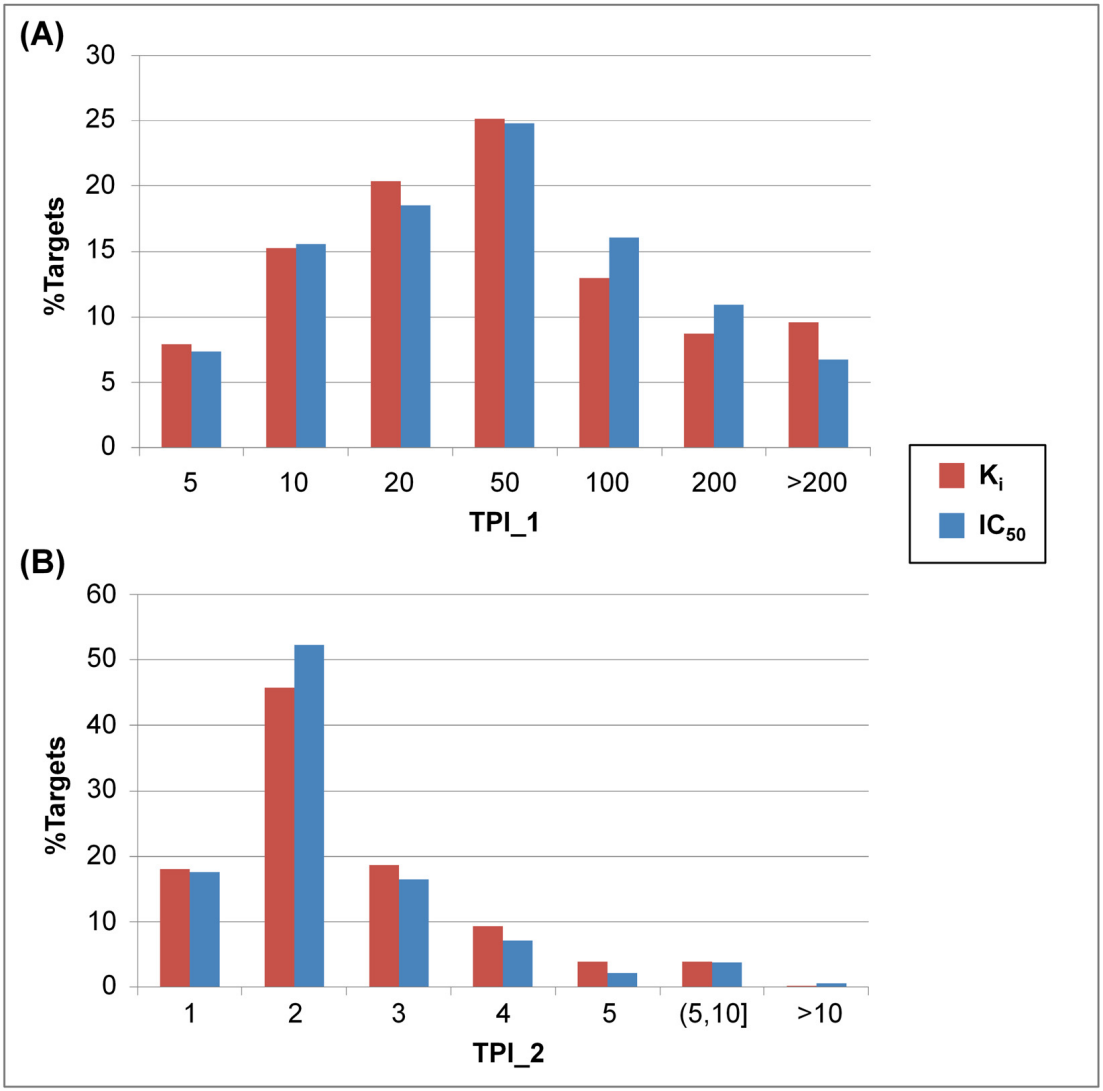
Distribution of target promiscuity indices. Shown is the distribution of (**A**) TPI_1 and (**B**) TPI_2 values for 354 targets from the K_i_ (red) and for 649 targets from the IC_50_ set (blue), respectively. For each of these targets, at least 10 active compounds were available.

For 354 (K_i_) and 649 targets (IC_50_) with at least 10 active compounds, the distribution of TPI_1 and TPI_2 values is reported in Fig 3. The value distributions were comparable for the K_i_ and IC_50_ sets. The TPI_1 value distribution in Fig 3A shows that the majority of targets had active compounds yielding more than 10 distinct scaffolds. The average TPI_1 value was 77 and 61 for the K_i_ and IC_50_ sets, respectively, indicating that many targets bound structurally diverse compounds (i.e., active compounds had many different core structures). Fig 3B shows the TPI_2 value distribution. Similar to previous studies reporting that ~35% of active database compounds had multi-target activity [1,7], our CPI calculations revealed that ~33% of compounds in the K_i_ but only 17% in the IC_50_ set were active against more than one target. The average CPI values were 1.6 (K_i_) and 1.3 (IC_50_).

In light of these findings, one might also anticipate obtaining comparably low TPI_2 values. Surprisingly, however, only ~18% of all targets interacted with compounds having exclusive single-target activity (i.e., producing a TPI_2 value of 1). By contrast, more than 80% of the targets interacted with one or more compounds having multi-target activity. For ~36% (K_i_) and ~30% (IC_50_) of the targets, TPI_2 values larger than 2 were obtained (with average TPI_2 values of 2.1 and 2.0 for the K_i_ and IC_50_ sets, respectively). Hence, essentially opposite promiscuity trends were observed for compounds and targets. Whereas the majority of compounds was only active against a single target, most targets bound varying numbers of promiscuous compounds.

### Comparison of TPI_1 and TPI_2 values

**Fig 4:**
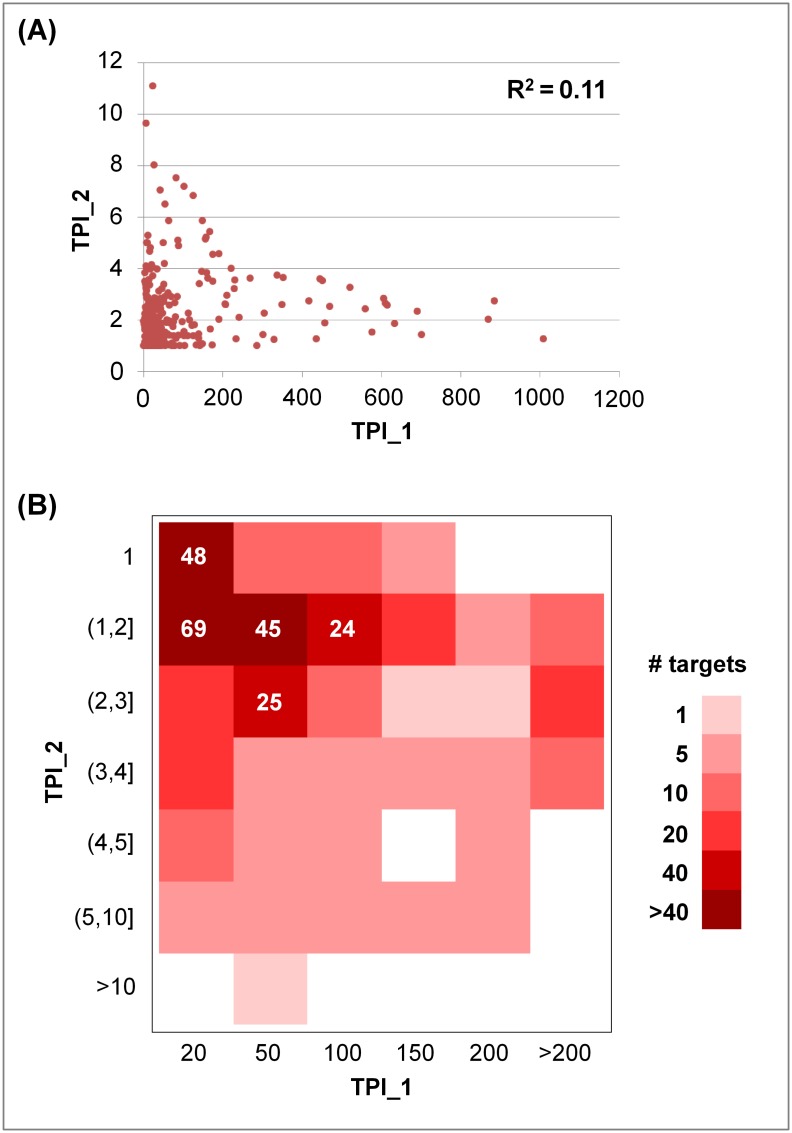
Comparison of promiscuity indices for targets in the K_i_ set. (**A**) For 354 targets from the K_i_ set, their TPI_1 and TPI_2 values are compared. Each dot in the scatter plot represents a target. The correlation coefficient (R^2^) for TPI_1 and TPI_2 values is provided. (**B**) Relationships between TPI_1 and TPI_2 values are captured in a heat map in which cells are colored according to the population density of targets. In addition, the number of targets is reported for cells that were populated with more than 20 targets using white numbers.

**Fig 5:**
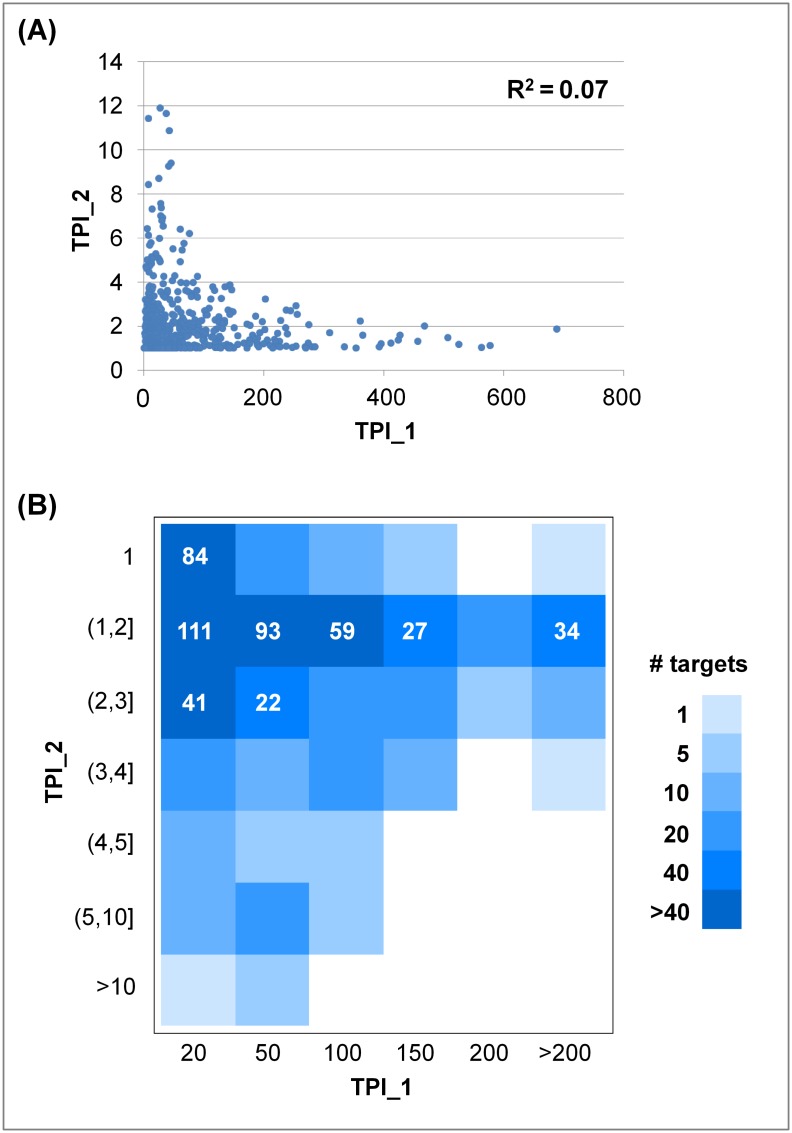
Comparison of promiscuity indices for targets in the IC_50_ set. For 649 targets from the IC_50_ set, their TPI_1 and TPI_2 values are compared. The representation is according to Fig 4.

Relationships between TPI_1 and TPI_2 values were further analyzed. As shown in Figs 4A and 5A for the K_i_ and IC_50_ sets, respectively, there was no apparent correlation between these two target promiscuity indices. Targets with TPI_1 values of less than 200 had a much broader distribution of TPI_2 values than targets with largest TPI_1 values (> 200). Furthermore, heat map representations of promiscuity index combinations were generated for targets from the K_i_ and IC_50_ sets, shown in Figs 4B and 5B, respectively. In these heat maps, rows represent seven ranges of TPI_2 values and columns six ranges of TPI_1 values. Each cell indicates the number of targets having corresponding TPI_1 and TPI_2 values. In addition, each row reflects the distribution of TPI_1 values for targets having comparable TPI_2 values and each column the distribution of TPI_2 values for targets having similar TPI_1 values.

For the K_i_ set (Fig 4B), targets interacting with compounds containing up to 200 distinct scaffolds displayed many different TPI_2 values covering five or six value ranges. The overall largest TPI_2 value (11.1) was observed for the histamine H2 receptor with a set of 26 antagonists (represented by 24 scaffolds). Hence, these antagonists were highly promiscuous. The majority of the targets produced low to intermediate TPI_2 values ranging from 1 to 3. The five most populated cells in the heat map contained 211 targets (i.e., ~60%). A subset of 117 targets with active compounds containing at most 20 scaffolds yielded TPI_2 values between 1 and 2.

Table 2 lists 10 exemplary targets from the K_i_ set that yielded the same or very similar TPI_1 values of varying magnitude but significantly different TPI_2 values. For example, compounds active against dihydroorotate dehydrogenase and NADPH oxidase 5 contained the same number scaffolds. However, inhibitors of dihydroorotate dehydrogenase had no other reported activities (TPI_2 value of 1.0), whereas all inhibitors of NADPH oxidase 5 had multi-target activity, resulting in a TPI_2 value of 3.4. In addition, for two related G protein coupled receptors (GPCRs; purinergic receptor P2Y12 and alpha-2c adrenergic receptor), known antagonists contained comparably large numbers of scaffolds (142 vs. 149), but their TPI_2 values differed significantly (1.0 vs. 5.9). Thus, purinergic receptor P2Y12 antagonists were exclusively active against this target, whereas 87.5% of the alpha-2c adrenergic receptor antagonists had multi-target activity.

**Table 2:** Targets with comparable TPI_1 and different TPI_2 values (K_i_ set).

ChEMBL Target ID	Target name	#Cpds	TPI_1	TPI_2	MT-Cpds
1966	Dihydroorotate dehydrogenase	17	5	1.0	0%
1926497	NADPH oxidase 5	15	5	3.4	100%
3232687	Apelin	13	10	1.0	0%
2938	Protein kinase C gamma	12	10	5.0	100%
4360	Monocarboxylate transporter 1	33	20	1.0	0%
1937	Histone deacetylase 2	32	20	4.0	100%
3717	Hepatocyte growth factor receptor	107	54	1.0	0%
1850	Dopamine D5 receptor	100	50	5.0	97.0%
2001	Purinergic receptor P2Y12	529	142	1.0	0%
1916	Alpha-2c adrenergic receptor	281	149	5.9	87.5%

Listed are 10 exemplary targets from the K_i_ set that yielded the same or very similar TPI_1 values (of varying magnitude) but significantly different TPI_2 values. For each target, its ChEMBL ID, name, and the number of active compounds (#Cpds) are provided together with TPI_1 and TPI_2 values. In addition, the percentage of compounds active against multiple targets (MT-Cpds) is given. “0%” means that all compounds only have reported activity against the given target but no others.

For the IC_50_ set (Fig 5B), observations similar to the K_i_ set were made. Four targets were identified that produced TPI_2 values greater than 10 including alpha-1d, -2b, and -2c adrenergic receptors and fibroblast growth factor receptor 3. Compounds active against these targets contained nine to 43 scaffolds. As reported in Table 3, a variety of targets were identified having the same or very similar TPI_1 but significantly different TPI_2 values.

**Table 3:** Targets with comparable TPI_1 and different TPI_2 values (IC_50_ set).

ChEMBL Target ID	Target name	#Cpds	TPI_1	TPI_2	MT-Cpds
1163101	Inositol-requiring protein 1	14	5	1	0%
2154	Group IIE secretory phospholipase A2	13	5	4.6	100%
3593	Lanosterol synthase	16	10	1	0%
3935	Serine/threonine-protein kinase Aurora-C	16	10	4.4	56.3%
1919	Voltage-gated calcium channel subunit alpha-2-1	37	25	1	0%
2056	Dopamine D1 receptor	44	26	8.7	77.3%
1921	Vasopressin V1b receptor	103	49	1	0%
287	Sigma opioid receptor	64	49	5.5	56.3%
5555	Acyl-CoA desaturase	300	144	1	0%
216	Muscarinic acetylcholine receptor M1	252	147	3.6	79.0%

Listed are 10 exemplary targets from the IC_50_ set that yielded the same or very similar TPI_1 values (of varying magnitude) but significantly different TPI_2 values. For each target, its ChEMBL ID, name, and the number of active compounds (#Cpds) are provided together with TPI_1 and TPI_2 values. In addition, the percentage of compounds active against multiple targets (MT-Cpds) is given. “0%” means that all compounds only have reported activity against the given target but no others.

Taken together, these results revealed that many different targets that recognized ligands with comparable degrees of structural diversity displayed markedly different tendencies to preferentially interact with selective or promiscuous compounds; a rather unexpected finding.

### Target family promiscuity

In light of these observations, the distribution of TPI_2 values was analyzed for 10 target families from the K_i_ set and 14 families from the IC_50_ set, which contained at least 10 targets each, as reported in Table 4. As discussed in the following, target families displayed very different promiscuity patterns.

**Table 4:** Target families.

Target family ID	Target family	#Targets	
		K_i_	IC_50_
59	Carbonic anhydrases	12	-
64	Chemokine receptors	11	10
73	Cysteine proteases	-	17
75	Cytochrome P450 isoforms	-	16
143	Histone deacetylases	-	11
165	Lipid-like ligand receptors	16	26
180	Metallo proteases	19	24
183	Monoamine receptors	35	30
208	Nuclear hormone receptors	12	21
222	PI3/PI4-kinases	-	10
234	Phosphodiesterases	-	11
275	Ser_Thr protein kinases	24	88
278	Serine proteases	28	20
281	Short peptide receptors	45	47
319	Tyr protein kinases	14	48

**Fig 6:**
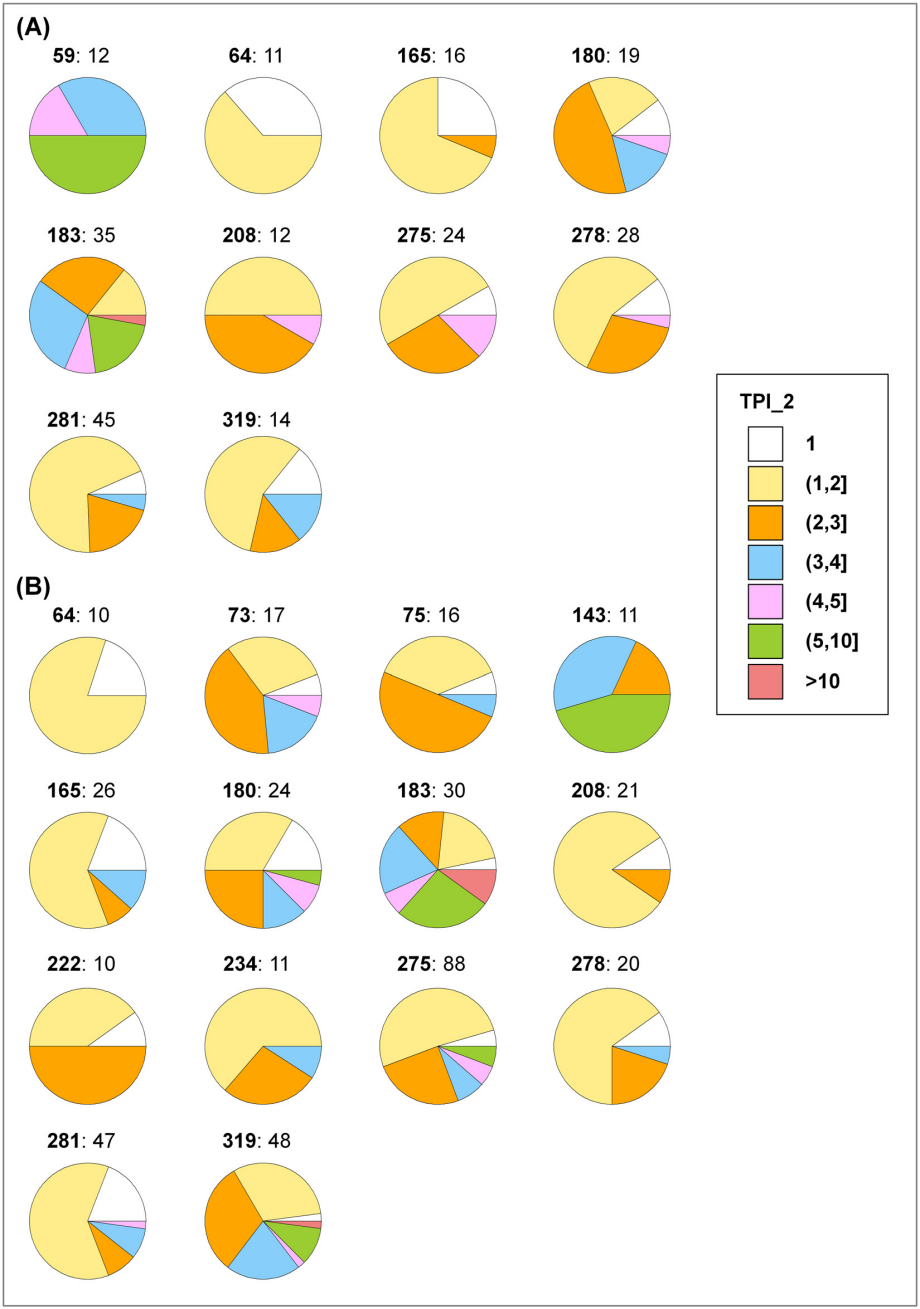
Target family promiscuity. The distribution of targets with varying TPI_2 values is reported in pie charts for (**A**) 10 target families from the K_i_ set and (**B**) 14 families from the IC_50_ set that contain at least 10 individual targets. Each color-coded pie chart segment reports the proportion of targets with TPI_2 values falling into a specific range. Seven value ranges are defined and colored-coded, as indicated on the right. For each family, an ID (bold) and the number of targets are provided. For example, “**59**: 12” means that family 59 contains 12 targets (K_i_ set). Target families are listed in Table 4.

Listed are 15 target families that contain 10 or more targets. For each family, its ID according to Fig 6 is given and the number of targets in the K_i_ and IC_50_ sets is reported. “-” indicates that there are fewer than 10 targets for the corresponding family in the K_i_ or IC_50_ set. For these families, the distribution of TPI_2 values is reported in Fig 6.

Fig 6 reports the intra-family distribution of TPI_2 values in a pie chart format. In Fig 6A (K_i_ set), three target families (ID 59, 64, and 208) that contained 11 or 12 targets yielded distinct intra-family TPI_2 distributions. For the chemokine receptor family (ID 64), ~1/3 of the targets only interacted with selective compounds (TPI_2 value of 1) while ~2/3 yielded TPI_2 values between 1 and 2, due to ligands with multi-target activity. For the nuclear hormone receptor family (208), no target was found to only interact with selective compounds. Fig 6B (IC_50_ set) reveals comparable results for four families (ID 64, 143, 222, and 234) with 10 or 11 targets including chemokine receptors, which displayed varying preferences for selective or multi-target compounds.

Several of the target families in Table 4 were closely related to each other including different GPCR, kinase, or protease families. For related families, different promiscuity patterns also emerged. For example, four GPCR families (ID 64, 165, 183, and 281) were associated with both the K_i_ and IC_50_ sets and showed different distributions of TPI_2 values. The degree of target promiscuity increased from the chemokine (64) over the lipid-like ligand (165) and short peptide (281) to the monoamine (183) receptor family. Hence, targets in these families showed an increasing tendency to bind promiscuous ligands. Furthermore, the serine/threonine (275) and tyrosine (319) kinase families displayed similar distributions of TPI_2 values for the IC_50_ set (Fig 6B) that notably differed from the PI3/PI4-kinase family (222).

Finally, the analysis of TPI_2 value distributions also identified target families with an overall strong preference to interact with promiscuous compounds including, for example, the carbonic anhydrase (Fig 6A; ID 59), histone deacetylase (Fig 6B; 143), or monoamine receptor family (Fig 6A and 6B; 183). In particular, histone deacetylases and monoamine receptors continue to be high-profile therapeutic targets and medicinal chemistry efforts are often heralded to identify new active compound classes for them. However, targets in these families are shown to display a strong tendency to recognize promiscuous compounds and are likely to be involved in many polypharmacological effects. The characteristics should be considered in the context of drug development.

## Conclusions

An intuitive methodological framework has been introduced to systematically explore target promiscuity. Although the exploration of polypharmacology has thus far mostly focused on compound promiscuity, differences in the ability of targets to interact with small molecules inevitably also make important contributions to the formation of polypharmacological networks. For our analysis of target promiscuity, simple first- and second-order target promiscuity indices were designed to quantify the tendency of targets to recognize structurally diverse and promiscuous compounds and relate these characteristics to each other. Care was taken to select high confidence activity data and target annotations as a basis for the analysis. Because assay-independent K_i_ and assay-dependent IC_50_ values cannot be directly compared, K_i_- and IC_50_-based data sets were separately generated and yielded similar results in promiscuity analysis. However, for compounds and targets, opposite promiscuity trends were detected. The majority of compounds were only active against a single target, whereas most targets bound varying numbers of promiscuous compounds. On the basis of TPI_1 calculations, many targets interacted with compounds representing different levels of scaffold diversity. TPI_2 calculations then revealed that many targets preferentially bound either selective or promiscuous compounds. Importantly, a variety of targets with ligands of comparable structural diversity displayed markedly different preferences to interact with compounds having single- or multi-target activity. This was also observed for targets capable of binding structurally highly diverse compounds. Furthermore, preferences for binding of selective vs. promiscuous compounds emerged at the level of target families that mostly interacted with promiscuous compounds. Structural features of targets or families that correlate with their propensity to interact with promiscuous vs. selective compounds are currently unknown, which provides opportunities for future research.

Taken together, the findings reported herein further improve our understanding of promiscuity at the level of targets and refine our view of the molecular basis of polypharmacology. In addition, through calculation and comparison of target promiscuity indices, as introduced herein, it can easily be estimated how likely it might be to identify selective compounds for a target of interest on the basis of available compound activity data for this and closely related targets. Furthermore, targets that are most likely to contribute to polypharmacology networks can also be identified via the same route. These practical applications should be of considerable interest in pharmaceutical research.
